# Curcumin decreases malignant characteristics of glioblastoma stem cells via induction of reactive oxygen species

**DOI:** 10.1186/s12885-017-3058-2

**Published:** 2017-02-04

**Authors:** Zachary C. Gersey, Gregor A. Rodriguez, Eric Barbarite, Anthony Sanchez, Winston M. Walters, Kelechi C. Ohaeto, Ricardo J. Komotar, Regina M. Graham

**Affiliations:** 1 Department of Neurosurgery, University of Miami Miller School of Medicine, Miami, Florida USA; 2 Department of Neurological Surgery, University of Miami Brain Tumor Initiative (UMBTI) Research Laboratory, Lois Pope LIFE Center, 2nd Floor, 1095 NW 14th Terrace, Miami, Florida 33136 USA

**Keywords:** Glioblastoma, Stem cell, STAT3, Curcumin, Reactive oxygen species, Brain tumor, Natural product

## Abstract

**Background:**

Glioblastoma Multiforme (GBM) is the most common and lethal form of primary brain tumor in adults. Following standard treatment of surgery, radiation and chemotherapy, patients are expected to survive 12–14 months. Theorized cause of disease recurrence in these patients is tumor cell repopulation through the proliferation of treatment-resistant cancer stem cells. Current research has revealed curcumin, the principal ingredient in turmeric, can modulate multiple signaling pathways important for cancer stem cell self-renewal and survival.

**Methods:**

Following resection, tumor specimens were dissociated and glioblastoma stem cells (GSCs) were propagated in neurosphere media and characterized via immunocytochemistry. Cell viability was determined with MTS assay. GSC proliferation, sphere forming and colony forming assays were conducted through standard counting methods. Reactive oxygen species (ROS) production was examined using the fluorescent molecular probe CM-H2DCFA. Effects on cell signaling pathways were elucidated by western blot.

**Results:**

We evaluate the effects of curcumin on patient-derived GSC lines. We demonstrate a curcumin-induced dose-dependent decrease in GSC viability with an approximate IC_50_ of 25 μM. Treatment with sub-toxic levels (2.5 μM) of curcumin significantly decreased GSC proliferation, sphere forming ability and colony forming potential. Curcumin induced ROS, promoted MAPK pathway activation, downregulated STAT3 activity and IAP family members. Inhibition of ROS with the antioxidant N-acetylcysteine reversed these effects indicating a ROS dependent mechanism.

**Conclusions:**

Discoveries made in this investigation may lead to a non-toxic intervention designed to prevent recurrence in glioblastoma by targeting glioblastoma stem cells.

**Electronic supplementary material:**

The online version of this article (doi:10.1186/s12885-017-3058-2) contains supplementary material, which is available to authorized users.

## Background

Glioblastoma multiforme (GBM) is the most common and deadly primary malignant brain tumor. GBM comprises about 15% of all intracranial tumors in adults ages 40–75 [1]. The tumor is exceptionally aggressive, with a mean survival of less than 15 months and a 5-year survival rate of 9.8% after standard therapy of resection, radiation and temozolomide chemotherapy [2, 3]. Despite numerous efforts, there has been stagnation in the advancement in treatment of this disease. The lack of improvement in survival rates of glioblastoma has led to the identification of novel therapeutic mechanisms such as targeting cancer stem cells (CSCs), also known as tumor initiating cells or cancer stem-like cells, in order to eradicate this lethal disease.

CSCs are small subset of cells within tumors that have stem-cell-like characteristics that allow them to sustain and repopulate the cancer [4]. The unique qualities of CSCs allow them to evade the chemotherapy and radiation that destroys the bulk of the tumor, eventually leading to the recurrence of disease. This idea has led researchers in search for targeted therapies that will eliminate CSCs and therefore prevent the relapse of cancer [4]. A compound that has shown promising anti-CSC properties is the natural phenol curcumin.

Curcumin is the principal curcuminoid in the Indian plant turmeric that has been used for thousands of years in Asian medicine to treat inflammatory conditions. Curcumin has also been shown to have antineoplastic properties including inhibition of proliferation, inducing apoptosis, inhibiting invasion and metastasis and decreasing angiogenesis in multiple tumors including glioblastoma [5–8]. Specifically, curcumin targets CSCs in vitro and in vivo in several cancers, including breast, colorectal, esophageal and glioma [9–13]. It is proposed that these effects are made through curcumin’s ability to induce reactive oxygen species [14–20].

Reactive oxygen species (ROS) are natural products formed by the metabolism of oxygen whose regulation plays an essential role in normal cell signaling and homeostasis [21]. The dysregulation of ROS has been implicated in many diseases such as dementia, cardiovascular disease, as well as cancer [22–24]. Current research also suggests that ROS have anti-neoplastic effects on CSCs and that these effects are brought about through the modulation of several molecular pathways including Mitogen-activated protein kinases (MAPKs) and Janus kinas (JAK)- Signal Transducer and Activator of Transcription (STAT3) signaling cascades [25–32]. Aberrations of the MAPKs and JAK-STAT3 pathways have been shown to be critical in the tumorgenesis and maintenance of GBM [33–37].

In this study, we assess the effects of curcumin on glioblastoma stem cells (GSCs) and propose the molecular mechanisms behind such effects.

## Methods

### Cells and cell culture

Human Glioblastoma Multiforme (GBM) tissue was obtained from five adult patients from the University of Miami Department of Neurosurgery diagnosed with WHO-IV gliomas based on the World Health Organization (WHO) classification of tumors of the Central Nervous System. Patients or guardians provided written informed consent prior to tumor sample retrieval. Samples were named Glio3, Glio4, Glio9, Glio11 and Glio14. GBM stem-like cell lines were generated as previously described [38]. Briefly, tumors were mechanically and enzymatically dissociated, red blood cells were removed using Red Cell Lysis buffer (SigmaAldrich, St. Louis, MO), Cells were filtered and plated in a 3:1 ratio of Dulbecco’s Modified Eagle’s medium (DMEM): F12 (Gibco, Carlsbad, Ca) media supplemented with 1% penicillin and streptomcycin (penn/strep), 20 ng/ml each of human epidermal growth factor and human fibroblast growth factor, and 2% Gem21 NeuroPlex Serum-Free Supplement (Gemini Bioscience, Sacramento, CA); a formulation consistent for the generation of neurospheres. The GBM cell lines U87, U251 and U235 were purchased from ATCC (Manassas, VA) and were maintained in RPMI media supplemented with 10% FBS and 1% penn/strep. These established GBM cell lines grew in an adherent fashion. All cell lines were routinely tested for mycoplasma using LookOut mycoplasma PCR detection kit (SigmaAldrich, St. Louis, MO) according to the manufacturer’s instructions and were maintained at 37 °C in a humidified 5% CO_2_ incubator.

### Immunofluorescence

To evaluate stem cell marker expression, neurospheres were dissociated mechanically or enzymatically with Accutase (Gemini Bioscience, Sacramento, CA). To facilitate adherence, cells were plated on poly-L-lysine/laminin coated four-well plates in neurosphere media. Cells were fixed in 4% paraformaldehyde, blocked and permeabilized with a 5% bovine serum albumin (BSA) with 0.6% Triton-× 100 and then treated with the primary antibodies Nestin (Abcam, Cambridge, MA), Sox2, Musashi 1, CD44, Bmi-1 (Cell Signaling Technology, Danvers, MA), CD133 (Biorbyt, Cambridge, UK) and A2B5 (A2B5 clone 105, ATCC, Manassas, VA). A “no primary control” was included for all antibodies tested for all cell lines. For these, the cells were incubated with only the antibody diluent (2.5% BSA, 0.3% triton, balance PBS). Cells were then treated with a fluorochrome-conjugated secondary antibody followed by Prolong Gold Antifade Reagent with DAPI (Thermo Fisher Scientific, Waltham, MA). Samples were examined under an EVOS FLoid Cell Imaging Station fluorescent microscope (Thermo Fisher Scientific, Waltham, MA).

### MTS assay

Viability was determined using the CellTiter 96® AQueous One Solution Cell Proliferation Assay (MTS) assay (Promega Madison, WI). Cells were seeded into 96-well plates using a modified neurosphere media containing 5% FBS at a density of 10,000 cells per well in 100 μl of cell culture media. Following treatment, media was aspirated and 100 μl of a 1:5 solution of MTS to cell culture media was added to each well and incubated for 1–4 h. Optical density was measured at 490 nm using BoiTek Synergy HT plate reader. To examine the effect of temozolomide (Sigma-Aldrich, St. Louis, MO), GBM stem cells were treated with 100 μM for 72 h or U87 cells were treated with 10–100 μM. Data is represented as the average of 3 separate experiments in which the viability was calculated as the percent of non-treated cells. To determine the effect of curcumin, cells were treated with increasing concentrations of curcumin (Sigma-Aldrich, St. Louis, MO) for 72 h. The IC_50,_ the concentration of curcumin at which 50% of cells were non-viable, was determined for a minimum of 3 separate experiments. Data is presented as the average IC_50_ for each cell line examined.

### Proliferation assay

To determine the effect on cell proliferation 100,000 cells were plated in 10 ml of neurosphere media (100 mm dish for Glio9, and T25 flask for Glio3). Curcumin was added at a concentration of 2.5 μM on day 0. Cells were counted on days 4, 7 and 10 using Orflo Technologies Cell Counter Moxi z (Ketchum, ID). Experiments were done in triplicate.

### Sphere forming assay

The effect of curcumin on clonogenic growth potential was determined using sphere-forming assays. Single cells were seeded at 50–100 cells per well in a 96-well plate and treated with 2.5 μM of curcumin on day 0. Spheres were manually counted under microscopy on day 14. All experiments were done in triplicate.

### Colony forming assay

Colony counting was performed to determine colony forming potential of the adherent GSC line. Cells were plated at 200 cells per well in 6-wells plates and treated with 2.5 μM of curcumin at day 0. Colonies were stained with 0.01% crystal violet (Sigma-Aldrich, St. Louis, MO) and counted under microscopy on day 14. Cell clusters of less than 50 cells were not considered colonies and therefore were not counted. Experiments were done in triplicate.

### ROS assay

Curcumin-induced ROS was visualized and quantitated using the general oxidative stress indicator CM-H2DCFDA (Thermo Fisher Scientific, Waltham, MA). CM-H2DCFDA passively diffuses into cells and reacts with ROS to yield a fluorescent adduct. For quantification, cells were split into 96-well plates in cell culture media with the addition of 5% FBS to cause adherence to the well bottoms. Samples were treated with 25 μM of curcumin in phenol red free media for 30 min, 4 h, and 24 h. Cells were incubated with 0.5 μM CM-H2DCFDA in PBS for 5 min subsequently washed in PBS and read at an excitation of 495 nm and an emission of 525 nm using BoiTek Synergy HT plate reader. Data is presented as fold change from non-treated cells. Curcumin-induced ROS activity was also examined using fluorescent microscopy. Dissociated GSCs were plated in neurosphere media on poly-L-lysine/laminin coated four-well plates. CM-H2DCFDA fluorescence was evaluated at 1, 6 and 24 h post curcumin (25 μM) treatment. Images were obtained using the EVOS FLoid Cell Imaging Station fluorescent microscope (Thermo Fisher Scientific, Waltham, MA).

### Western blot analysis

Neurospheres cultures, Glios 3, 4, 11 and 14 were plated and treated as neurospheres ranging in size from 100–300 μm as determined by light microscopy. At 8 or 24 h of treatment, the effect of curcumin, N-acetylcysteine (NAC, Sigma-Aldrich, St. Louis, MO) or the combination of curcumin and NAC on protein levels was determined by western blot analysis.

Our method for western blot analysis has previously described [39]. Briefly, GSCs were lysed in RIPA buffer, protein concentrations determined by using BCA protein assay and 20 μg of protein was loaded onto 8, 12 or 15% polyacrylamide gel (BioRad Hercules, CA) gels for electrophoresis and subsequently transferred onto nitrocellulose membranes. The membranes were then blocked for 1 h in 5% non-fat milk (Biorad, Hercules, CA) at room temperature (RT) and incubated with the primary antibody diluted in 2.5% BSA overnight. All primary antibodies were purchased from Cell Signaling (Danvers, MA) except for alpha-tubulin, which was purchased from Abcam (Cambridge, UK) and STAT3, which was purchased from Santa Cruz Biotechnology (Dallas, TX). Membranes were then incubated at room temperature with anti-mouse or anti-rabbit secondary antibodies for 1 h. Blots were developed using SuperSignal™ West Pico Chemiluminescent Substrate (Thermo Scientific Waltham, MA).

### Statistical analysis

Significance was determined using Student’s t-tests for all pairwise comparisons of the different treatments that were tested. The results are presented as the mean ± standard error mean (SEM). Significance was set at *p* < 0.05.

## Results

### Human GBM-derived cell lines display cancer stem cell characteristics

**Fig. 1:**
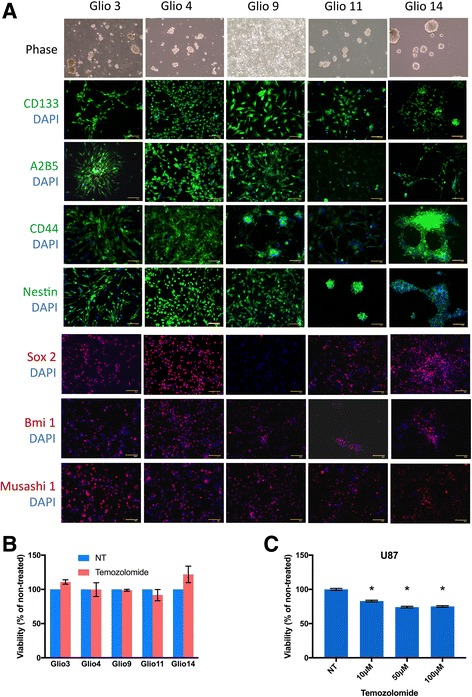
Patient-derived GBM Stem Cells and Characterization of GBM Stem Cell Lines. **a** Glio 3, 4, 9, 11, 14 immunostaining. Cells are positive for stem cell markers CD133, A2B5, CD44, Nestin, SOX2, Bmi 1 and musashi. Cell nuclei were counterstained with DAPI. Scale bar: 100 μm. **b** GBM stem cell lines were treated with100μm temozolomide and viability determined after 72 h with MTS assay. Results displayed as percent viable cells compared to untreated controls. **c** U87 cells were treated with temozolomide at concentrations shown and viability determined at 72 h with MTS assay. *p < 0.001 compared to non-treated controls (NT)

In neurosphere media four out of five cell lines formed spheres, where as the Glio9 grew in an adherent fashion (Fig. 1a). Since there is no definitive marker for GBM stem cells, we examined the expression of multiple putative cancer stem cell markers by immunocytochemistry [40–45]. Except for Glio9 the cell lines demonstrated expression of all markers examined (Fig. 1a). Negative controls for each antibody are shown in Additional file 1: Figure S1A. No SOX2 expression was observed in Glio9. Recently it has been shown that GBM stem cells can be further classified into subgroups, proneural and mesenchymal. These differ both morphologically (neurosphere verse a more adherent phenotype) and in stem cell marker expression [46]. The adherent fashion and the lack of SOX2 expression suggests that glio9 falls into the mesenchymal subgroup. In order to determine if our patient derived cell lines exhibited the cancer stem cell property of chemoresistance [47], we treated five cell lines with 100 μM temozolomide, the chemotherapeutic agent of choice for GBM. We chose a concentration of 100 μM since this is well above the reported (approximately 10 μM) peak levels in cerebral spinal fluid and brain tissue of treated GBM patients [48, 49]. Our results demonstrate that temozolomide had no significant effect on the viability of these GBM cell lines compared to non-treated controls (Fig. 1b). In contrast, the non-GBM stem cell line U87 was sensitive to temozolomide treatment at doses as low as 10 μM, the lowest dose examined (Fig. 1c). These data suggest that our patient-derived GBM cell lines demonstrate progenitor cell properties consistent with glioblastoma stem cells (GSCs).

### Curcumin decreases viability of glioblastoma stem cells and non-stem cells

**Fig. 2:**
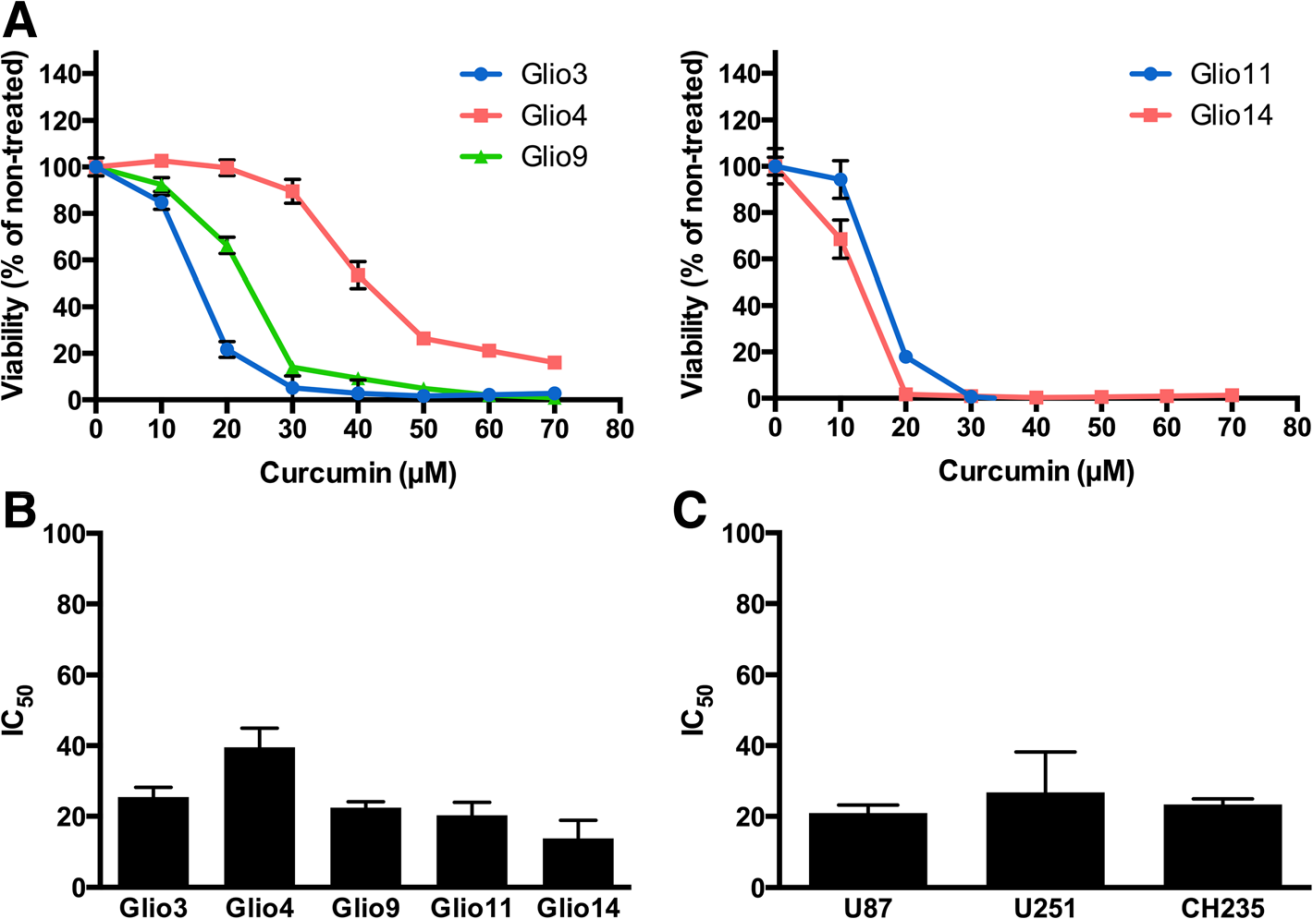
Effect of curcumin on GBM Stem Cell Lines and non-stem Cell Lines. **a** GBM stem cells were treated with increasing concentrations of curcumin and viability was assessed 72 h later with MTS assay. **b** MTS viability assay was used to determine concentrations needed to induce 50% cell death (IC_50_) in GBM stem cell lines. **c** MTS viability assay was used to determine concentrations needed to induce 50% cell death (IC_50_) in GBM non-stem cell lines

Several reports have demonstrated that curcumin has anti-neoplastic effects on glioblastoma cells [9, 50–52]. To determine the effect of curcumin on GSC viability we treated five GSC cell lines with increasing concentrations of curcumin for 72 h. In all cell lines analyzed, curcumin demonstrated a does-dependent decrease in viability (Fig. 2a). All cell lines reached levels less than 20% viability at 70 μM curcumin—the highest concentration tested. The concentration of curcumin at which 50% of cells were non-viable is known as the IC_50_. The IC_50_s were as follows: Glio3 25.5 μM (SEM: 2.7 μM), Glio4 39.5 μM (SEM: 5.4 μM), Glio9 22.5 μM (SEM: 1.7 μM), Glio11 20.3 μM (SEM: 3.7 μM), and Glio14 13.9 μM (SEM: 5.0 μM) (Fig. 2b). We also verified that curcumin decreases the viability of GBM non-stem cells using the established GBM cell lines U87, U251 and CH235. The IC_50_s of these common GBM cell lines were 30.0 μM (SEM: 2.2 μM) for U87, 26.8 μM (SEM: 11.5 μM) for U251, and 23.4 μM (SEM: 1.6 μM) for CH235 (Fig. 2c). Taken together, these results show that curcumin has a does-dependent effect on the viability of both GBM stem cells and non-stem cells.

### Curcumin inhibits proliferation, sphere-forming ability and colony-forming potential of glioblastoma stem cells

**Fig. 3:**
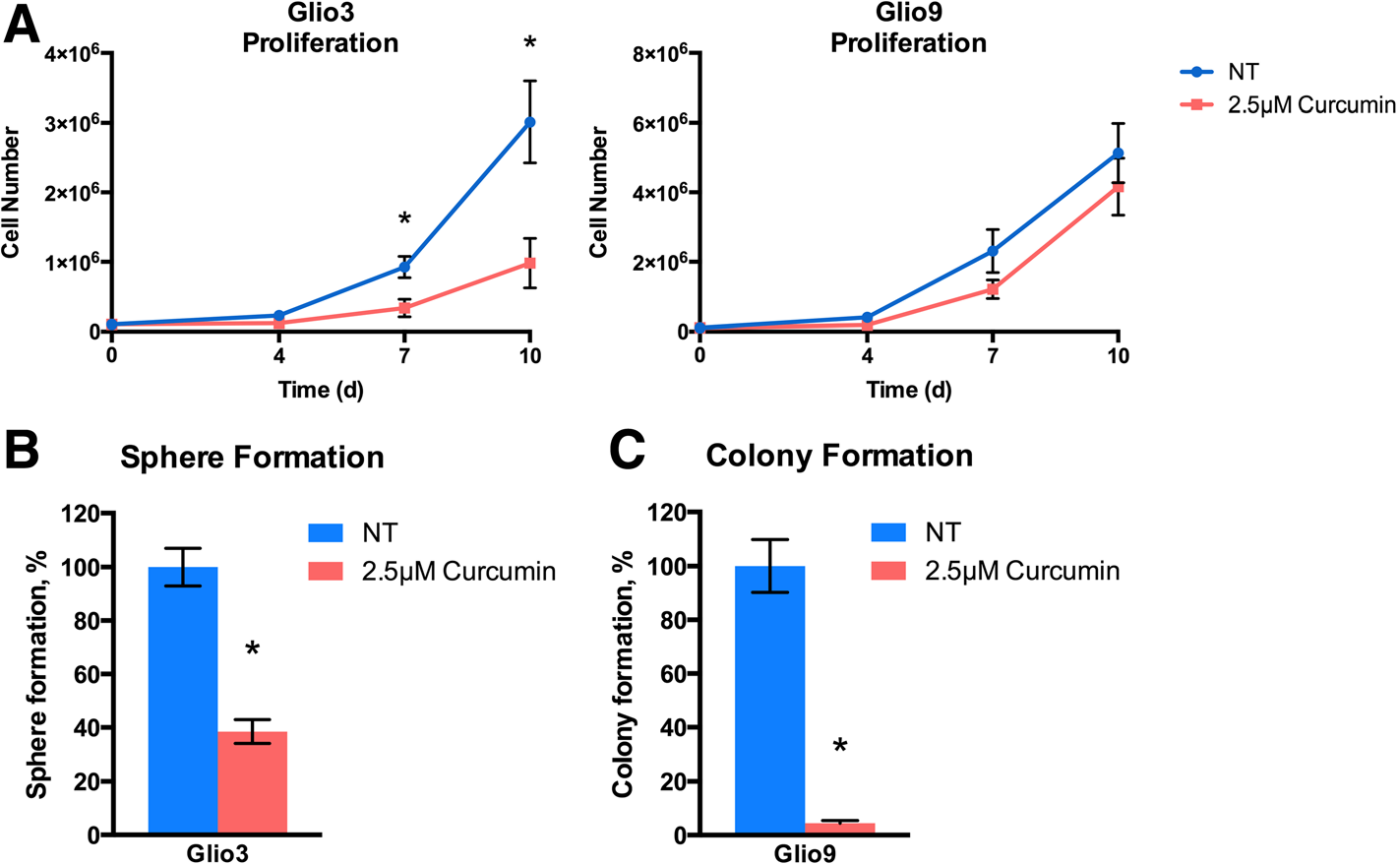
Curcumin decreases proliferation, sphere forming ability and colony forming potential in GSC cell lines. **a** Glio3 and Glio9 GSCs were plated at 1x10^5^ cells initially and treated with 2.5 μM curcumin on day 0. Cells were counted using Orflo Technologies Cell Counter Moxi z on days 4, 7 and 10. **b** Glio3 GSCs were seeded at 50–100 cells per well in a 96-well plate and treated with 2.5 μM curcumin on day 0. Spheres were counted on day 14. **c** Glio9 GSCs were plated at 200 cells and treated with 2.5 μM curcumin at day 0. Colonies were stained with crystal violet and counted on day 14. *p < 0.05, non-treated controls (NT) vs. curcumin treated

Cancer stem cells are marked by their ability to proliferate indefinitely and by their sphere- and colony-forming potential at the single cell level in vitro [53, 54]. We chose to carry out the remainder of the experiments in this study using Glio3, a non-adherent GSC cell line, and Glio9, an adherent GSC cell line, due to their similar IC_50_s and differing adherence patterns. In order to determine if curcumin affects the proliferative ability of GSCs, we plated Glio3 and Glio9 at 1×10^5^ cells and treated with 2.5 μM curcumin on day 0. Curcumin treated Glio3 showed a statistically significant decrease in cell number on days 7 and 10 (*p* < 0.05) compared to non-treated controls, whereas Glio9 showed a non-significant decrease in cell number on days 7 and 10 (Fig. 3a). To investigate whether curcumin has an effect on the sphere-forming capacity of GSCs, we seeded the non-adherent cell line Glio3 at 50–100 cells per well and treated it with 2.5 μM curcumin on day 0. Spheres were counted on day 14. Glio3 demonstrated a 60% decrease in sphere formation when treated with curcumin compared to non-treated controls (*p* <0.05) (Fig. 3b). The adherent cell line Glio9 was used to determine if curcumin affects the colony-forming ability of GSCs. Glio9 was plated at 200 cells per well and 2.5 μM curcumin was treated at day 0. On day 14, the curcumin treated cells showed a dramatic 95% reduction in colony number compared to non-treated controls (*p* < 0.05) (Fig. 3c). These data show that low doses of curcumin inhibit proliferation, sphere-forming and colony-forming potentials of GSCs.

### Curcumin induces ROS in glioblastoma stem cells

**Fig. 4:**
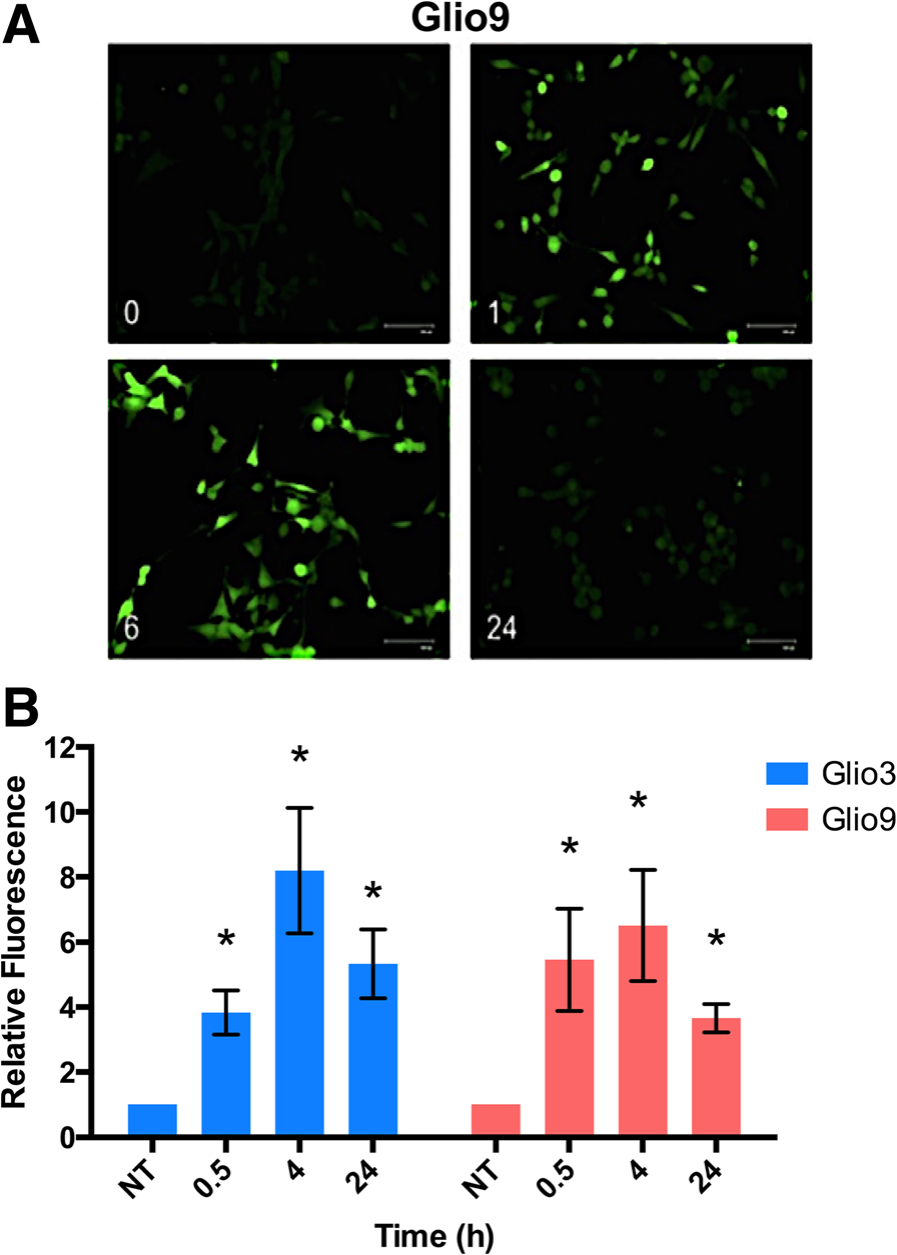
Curcumin induces reactive oxygen species activation in GSCs. **a** Curcumin-mediated ROS induction in the GSC glio9 was visualized using CM-H2DCFDA, which produces s a fluorescent adduct (*green*) in the presence of ROS, at 0, 1, 6 and 24 h under fluorescent microscopy. **b** ROS induction in the GSC glio3 and glio9 at 0, 0.5, 4 and 24 h following curcumin treatment was determined by measuring CM-H2DCFDA fluorescent intensities in a microplate reader. Data expressed as fold change over non-treated (NT) controls. *p < 0.05 compared to NT

Curcumin has been demonstrated to induce reactive oxygen species (ROS) in various cancer cell lines [55–57]. To determine if curcumin has the same effect on GSCs we used the molecular probe CM-H2DCFDA, a general oxidative stress indicator, to measure ROS via fluorescence in two cell lines. Under fluorescence microscopy, Glio9 showed an induction of ROS at the 1 and 6 h time points after treatment with 25 μM curcumin with a return to control levels at 24 h (Fig. 4a). After quantification, a one time treatment of 25 μM curcumin was shown to significantly induce ROS in Glio3 and Glio9 with a peak increase of approximately 6–8 fold relative fluorescence at 4 h post-treatment relative to non-treated controls (*p* < 0.05). ROS were shown to decrease 24 h post-treatment (Fig. 4b). These data suggest that curcumin may cause its effects in GSCs via induction of ROS.

### Curcumin induces MAPK activation, inactivates STAT3 and downregulates the STAT3 downstream target Survivin in glioblastoma stem cells

**Fig. 5:**
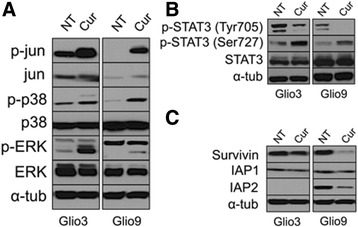
The effects of curcumin on molecular pathways. **a** Expression of p-jun, jun, p-p38, p38, p-ERK and ERK were assessed by western blot analysis in non-treated (NT) GSCs and 8 h after 25 μM of curcumin. **b** Expression of p-STAT3 (Tyr705), p-STAT3 (Ser727) and STAT3 was assessed by western blot analysis in non-treated GSCs (NT) GSCs and 8 h after 25 μM of curcumin. **c** Expression of the anti-apoptosis proteins Survivin, IAP1 and IAP2 were assessed in non-treated GSCs and 24 h after 25 μM of curcumin. Alpha-tubulin was used as a loading control for experiments **a**–**c**

Studies have demonstrated that ROS can induce the activation of multiple signaling pathways including the MAPK pathways in several cell types [58, 59]. We used western blot analysis to determine curcumin’s, and potentially ROS activation’s, modulation on different signaling pathways. Following 8 h of 25 μM curcumin treatment, the phosphorylated (activated) form of ERK, p38 and c-jun (as an indicator of JNK activation) was increased in the GSCs Glio3 and Glio9 (Fig. 5a). This was also demonstrated in all other GSC cell lines (Additional file 2: Figure S2), ERK has been shown to cause the repression of STAT3 activity via dephosphorylation at the Tyr705 position and phosphorylation at the Ser727 location [60]. Here we show that treatment with curcumin decreases the Tyr705 phosphorylated form of STAT3 and increases the Ser727 form in Glio3 and Glio9 (Fig. 5b). When STAT3 is dephosphorylated at the Tyr705 position and phosphorylated at the Ser727 position it is rendered inactive and is incapable of translocating to the nucleus to carry out its downstream effects. We also demonstrate the decreased expression of STAT3’s downstream target Survivin as well as the other anti-apoptosis proteins IAP1 and IAP2 (Glio9 only) in these GSCs (Fig. 5c). These results suggest that curcumin induces the activation of MAPKs and the inhibition of STAT3 activity in GSCs.

### N-acetylcysteine rescues curcumin-induced effects on glioblastoma stem cells

**Fig. 6:**
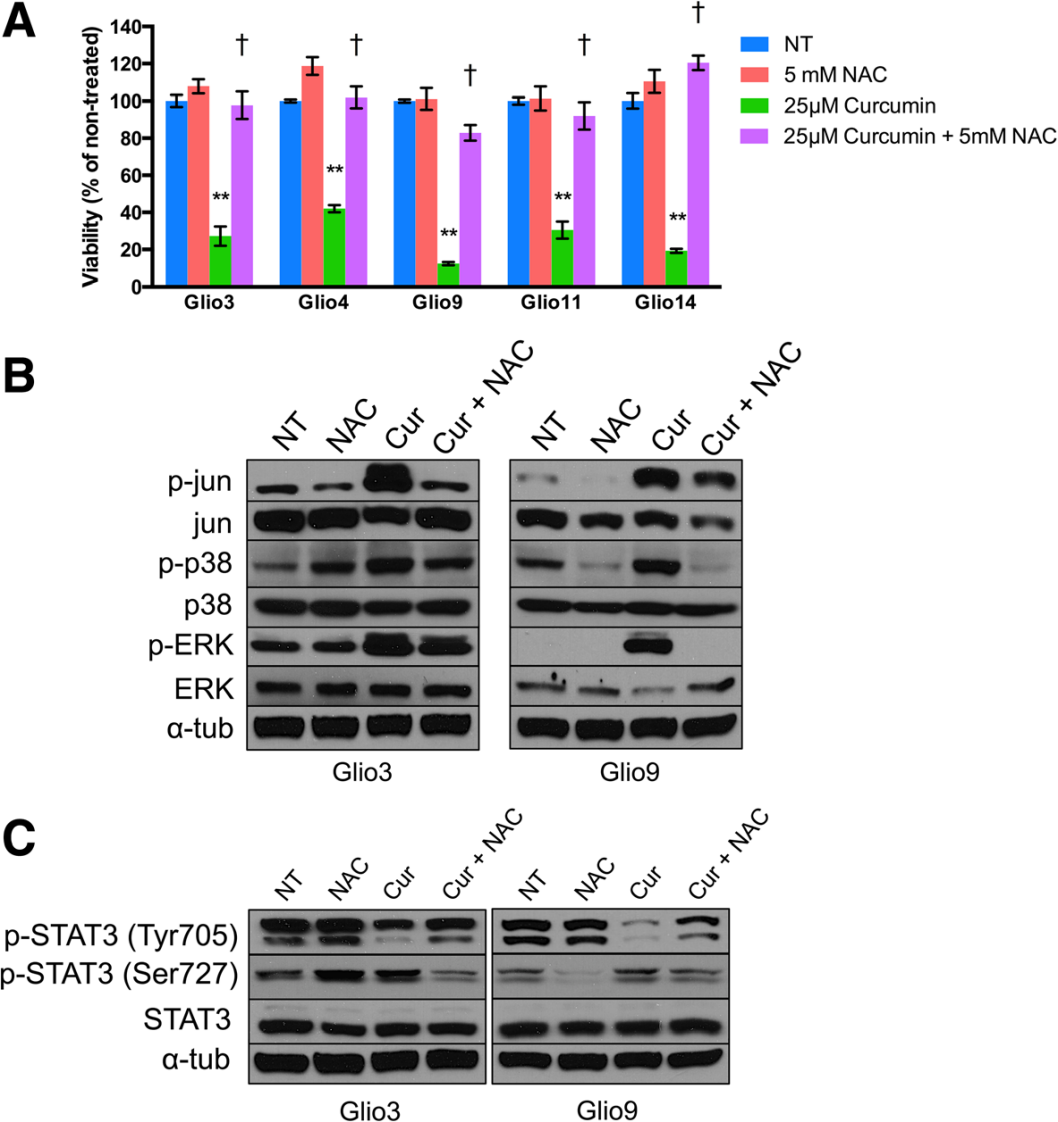
N-acetylcysteine (NAC) rescues curcumin-induced decrease in viability and modulation of molecular pathways in multiple GSC cell lines. **a** GSCs were treated with 5 mM NAC alone, 25uM curcumin alone or 5 mM NAC and 25uM curcumin in combination. Viability was assessed at 72 h using the MTS assay. Results displayed as percent viable cells compared to untreated controls (NT). **b** Expression of p-jun, jun, p-p38, p38, p-ERK and ERK was assessed in non-treated (NT), 5 mM NAC treated, 25 μM curcumin treated, and pretreated 5 mM NAC followed by 25 μM curcumin treated GSCs after 8 h. **c** Expression of p-STAT3 (Tyr705), p-STAT3 (Ser727) and STAT3 was assessed in non-treated (NT), 5 mM NAC treated, 25 μM curcumin treated, and pretreated 5 mM NAC followed by 25 μM curcumin treated GSCs after 8 h. Alpha-tubulin was used as a loading control. **P < 0.001 vs. NT. †*P* < 0.001 vs. 25 μM Curcumin

N-acetylcysteine (NAC) is an antioxidant shown to decrease ROS [61, 62]. To test whether ROS induction was truly the mechanism for curcumin’s anti-malignant effects on GSCs, we conducted a cell viability assay and western blot analysis to determine if NAC could rescue curcumin’s effects on GSCs. We treated cells with 5 mM NAC, 25 μM curcumin, and a combination of both treatments and viability was determined at 72 h. Treatment with NAC alone had no significant effect on viability on all cell lines except for Glio4, which showed an 18.7% increase in viability (*p* < 0.05). Treatment with curcumin alone showed significant decreases in viability in all cell lines compared to non-treated controls (*p* < 0.001). When cells were pretreated with NAC to prevent ROS induction, cell viability was significantly rescued in all cell lines compared to curcumin only treated cells (*p* < 0.001) (Fig. 6a). To determine if NAC treatment reverses curcumin’s effects on signaling pathways in Glio3 and Glio9, cells were treated with 5 mM NAC, 25 μM curcumin, and a combination of both treatments for 8 h. Western blot analysis indicates that NAC reversed the curcumin-induced MAPK activation (Fig. 6b) and STAT3 deactivation—signified by an increase in p-STAT3 (Tyr705) and a decrease in p-STAT3 (Ser727) (Fig. 6c). This was also demonstrated in all GSC cell lines at the Tyr705 position (Additional file 3: Figure S3). These data demonstrate that ROS induction may be the mechanism behind curcumin’s anti-cancer effects.

## Discussion

A growing body of evidence indicates that GSCs are responsible for tumor formation, progression and recurrence and that targeting these cells may be paramount in the eradication of GBM [63, 64]. Studying GSCs from patient derived GBM samples is the best model of disease in humans, as it has been shown that established, indefinitely passaged GBM cell lines do not predict clinical drug efficacy and are not representative of patient tumors [65]. Here we demonstrate the anti-neoplastic effects of curcumin, a blood brain barrier permeable compound shown to be non-toxic to normal astrocytes and neurons, on patient derived GSCs [66, 67].

In this study we demonstrate through a neurosphere growth pattern (with the exception of the adherent Gio9), chemoresistance and the expression of all tested stem cell markers (with the exception of SOX2 in Glio9) in all cell lines that our samples are indeed GSCs (Fig. 1). Due to its adherent nature and lack of SOX2 expression, we hypothesize that Glio9 is of the mesenchymal GBM subtype [46, 68]. We show that curcumin decreases viability of GSCs in a dose dependent manner (Fig. 2) and that low doses of curcumin inhibit proliferation, sphere formation and colony formation of GSCs (Fig. 3). Experiments at doses this low are lacking from the GBM literature. We have shown that treatment with curcumin induces ROS activity (Fig. 4) and that pretreatment with the antioxidant n-acetylcysteine reverses curcumin’s effects on viability and molecular pathways (Fig. 6). It has been shown that the ERK pathway is inducible through ROS [58, 59] and that activated ERK can cause repression of STAT3 and downregulation of its downstream targets though an inhibition of its tyrosine 705 phosphorylation and activation of its serine 727 phosphorylation [60]. Although more work needs to be done, our data suggests that curcumin may exert its effects through this mechanism via induction of ROS.

The role of ROS in cancer is dichotomist in nature. Low levels of ROS have been shown to promote cancer through stimulation of cell proliferation, increased cell survival and amplified angiogenesis through activation of several pathways including NF-κB [69–71]. High levels of ROS have been shown to have anti-cancer effects by inducing cell cycle arrest and apoptosis via several mechanisms including Rac-1/NADPH oxidase pathway induction [72, 73]. CSCs have been shown to have lower intracellular ROS content due to increased expression of free radical scavenging systems [74]. Although this may indicate CSC ROS resistance, several studies have demonstrated ROS-induced targeting of CSCs. Induction of ROS through niclosamide treatment in AML, parthenolide treatment in AML and CML, and arsenic trioxide treatment in PML (promyelocytic leukemia) target CSCs [75–77]. In this study we demonstrate that curcumin-induced ROS targets glioblastoma stem cells.

Curcumin has been shown to be an effective CSC targeting molecule in glioma as well as other tumor types [9–12] while maintaining a minimal side effect profile even at high doses of 12 g/day [78]. The main hurdle facing curcumin as a potential chemotherapeutic agent is its bioavailability [14]. When dosed orally, unformulated curcumin has been shown to reach peak plasma levels of <2 μM in humans [79]. In order to overcome this limitation, researchers have formulated several bioavailable forms of curcumin. Nano-emulsion curcumin, thermacurcumin (curcumin within colloidal nanoparticles), and curcumin within N-trimethyl chitosan coated solid lipid nanoparticles have been shown to reach peak plasma levels of 12.6 μM, 4.6 μM, and 3.28 μM respectively in rodent models [80–82]. In this study we demonstrate that 2.5 μM of curcumin inhibits the self-renewal properties of GSCs. In order to target GSC viability at curcumin levels of 25 μM (Fig. 6) and above, alternative routes of administration must be considered. Polymeric drug and convection-enhanced delivery systems have been shown to deliver high local concentrations of active agents while decreasing systemic toxicities in GBM and may serve to circumvent the bioavailability issues facing curcumin [83]. Currently curcumin is being evaluated clinically for neurological diseases including bi-polar disorder and Alzheimer’s disease as well as for multiple cancers, however clinical trials are needed to determine the potential of curcumin alone and in combination with radiotherapy and or chemotherapy for GBM patients.

## Conclusions

In summary, we have found that curcumin targets glioblastoma stem cells though the induction of ROS, potentially through downregulation of STAT3 activity. The importance of STAT3 in GBM has previously been described [84]. Specifically, inhibition of STAT3 signaling decreased GSC survival both in culture and in orthotopic xenograft models [85]. Furthermore, levels of STAT3’s downstream target, Survivin correlate with astrocytoma grade and may be predictive of poor patient survival [86, 87]. We show that low doses of curcumin inhibit the self-renewal properties of GSCs—an important characteristic for a chemotherapy targeting GBM relapse—and that curcumin decreases GSC viability in a dose dependent manner. These findings indicate that curcumin may be a safe future chemotherapeutic agent for the treatment of glioblastoma and further studies are warranted.

## Additional files

Additional file 1:
**Figure S1.** No primary controls for stem cell immunofluorescence shown in Fig. 1a. For control staining, antibody diluent without primary antibody was used, followed by the secondary antibody. Cells were counterstained with DAPI to identify nucleus. No stem cell marker fluorescence was observed in control cells. Scale bar: 100 μm. (TIFF 1896 kb)
Additional file 2:
**Figure S2.** The effects of curcumin on MAPKs in additional GBM stem cell lines. Expression of p-jun, jun, p-p38, p38, p-ERK and ERK were assessed by western blot analysis in non-treated (NT) GSCs and 8 h after 25 μM of curcumin. Alpha-tubulin was used as a loading control for all experiments. (TIFF 562 kb)
Additional file 3:
**Figure S3.** N-acetylcysteine (NAC) rescues curcumin-induced p-STAT3 (Tyr705) activation in additional GBM stem cell lines. Expression of p-STAT3 (Tyr705) and STAT3 was assessed in non-treated (NT), 5 mM NAC treated, 25 μM curcumin treated, and pretreated 5 mM NAC followed by 25 μM curcumin treated GSCs after 8 h. Alpha-tubulin was used as a loading control. (TIFF 345 kb)

## Abbreviations

AML: Acute myeloid leukemia
ATCC: American type culture collection
BCA: Bicinchoninic acid
BSA: Bovine serum albumin
CML: Chronic myeloid leukemia
DAPI: 4′,6-diamidino-2-phenylindole
DMEM: Dulbecco’s modified Eagle’s medium
ERK: Extracellular signal–regulated kinases
FBS: Fetal bovine serum
GBM: Glioblastoma multiforme
GSCs: Glioblastoma stem cells
IAP: Inhibitor of apoptosis protein
JAK: Janus kinase
JNK: Jun N-terminal kinases
MAPK: Mitogen-activated protein kinases
NAC: N-acetylcysteine
NADPH: Nicotinamide adenine dinucleotide phosphate
NF-κB: Nuclear factor kappa-light-chain-enhancer of activated B cells
PCR: Polymerase chain reaction
PML: Promyelocytic leukemia
Rac-1: Ras-related C3 botulinum toxin substrate 1
ROS: Reactive oxygen species
RPMI: Roswell Park Memorial Institute
SEM: Standard error of the mean
STAT3: Signal transducer and activator of transcription 3
WHO: World health organization
