# Supplementary figures and images for: Curcumin decreases malignant characteristics of glioblastoma stem cells via induction of reactive oxygen species
Source: BMC Cancer. 2017 Feb 4;17:99. doi: 10.1186/s12885-017-3058-2 (PMC5292151; doi:10.1186/s12885-017-3058-2)

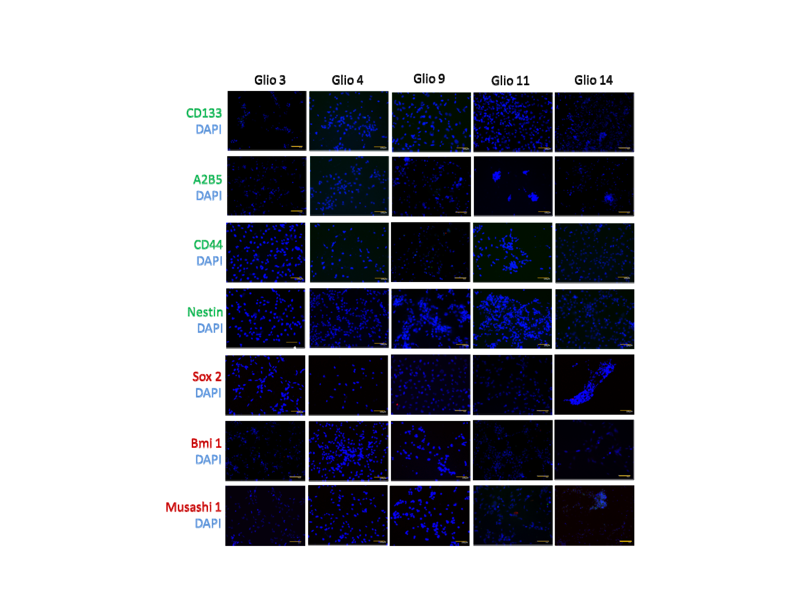

Supplement: Additional file 1: — Figure S1. No primary controls for stem cell immunofluorescence shown in Fig. 1a. For control staining, antibody diluent without primary antibody was used, followed by the secondary antibody. Cells were counterstained with DAPI to identify nucleus. No stem cell marker fluorescence was observed in control cells. Scale bar: 100 μm. (TIFF 1896 kb) [file 12885_2017_3058_MOESM1_ESM.tiff]

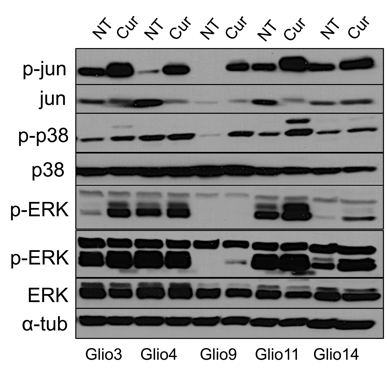

Supplement: Additional file 2: — Figure S2. The effects of curcumin on MAPKs in additional GBM stem cell lines. Expression of p-jun, jun, p-p38, p38, p-ERK and ERK were assessed by western blot analysis in non-treated (NT) GSCs and 8 h after 25 μM of curcumin. Alpha-tubulin was used as a loading control for all experiments. (TIFF 562 kb) [file 12885_2017_3058_MOESM2_ESM.tiff]

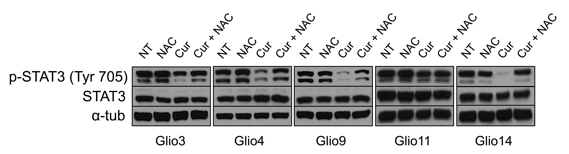

Supplement: Additional file 3: — Figure S3. N-acetylcysteine (NAC) rescues curcumin-induced p-STAT3 (Tyr705) activation in additional GBM stem cell lines. Expression of p-STAT3 (Tyr705) and STAT3 was assessed in non-treated (NT), 5 mM NAC treated, 25 μM curcumin treated, and pretreated 5 mM NAC followed by 25 μM curcumin treated GSCs after 8 h. Alpha-tubulin was used as a loading control. (TIFF 345 kb) [file 12885_2017_3058_MOESM3_ESM.tiff]
